# Sigmoid atresia: Case report and literature review

**DOI:** 10.1016/j.ijscr.2024.109434

**Published:** 2024-02-23

**Authors:** Ahmed Abokrecha, Ahmed Gamal Sayed, Khalid Alnajjar, Orjuwan Ayidh Almatrafi, Maeen Aldamouni

**Affiliations:** a Department of Pediatric Surgery, Maternity and Children Hospital, Makkah, Saudi Arabia; b College of Medicine, Alfaisal University, Riyadh, Saudi Arabia; c College of Medicine, Um Alqura University, Mecca, Saudi Arabia

**Keywords:** Sigmoid atresia, Congenital anomaly, Intestinal obstruction, Case report

## Abstract

**Introduction and importance:**

Intestinal atresia is a congenital anomaly commonly happening in the small bowel and rarely in the colon. Colonic atresia can manifest as intestinal obstruction with abdominal distention and bilious vomiting.

**Case presentation:**

A 3-day-old male new-born who was referred from a rural hospital, full term, product of normal vaginal delivery, with a weight of 2400 g. The patient had a complaint of bilious vomiting, inability to pass meconium, and abdominal distension for three days. On clinical examination the patient had visible bowel loops and yellowish aspiration from the orogastric tube. An erect abdominal radiograph showed distended bowel loops and sharp air-fluid levels. Administration of contrast enema revealed resistance to pass the rectal tube and stopping of contrast pas the rectosigmoid region with contrast spillage from the anus.

**Clinical discussion:**

Sigmoid atresia is a rare congenital anomaly that occurs in approximately 1 in 20,000 live births. The most common presentation is bilious vomiting and abdominal distension in the first 24 h of life. Diagnosis is confirmed with abdominal X-ray and contrast enema. Treatment is surgical, with primary repair being the most common approach. He underwent two stages of repair with an end colostomy and mucous fistula, then closure of the colostomy after four months of the first stage.

**Conclusion:**

This presentation requires clinical suspicion with prompt care, as this condition can mimic Hirschsprung's disease. In our case, the patient has a classical presentation of sigmoid atresia that had an early diagnosis, which resulted in a better outcome.

## Highlights

• Colonic atresia is considered one of the rarest atresia of the gastrointestinal tract.
• Sigmoid atresia should be differentiated from other lower GIT congenital causes of obstruction.
• Sigmoid atresia needs urgent intervention through primary or staged repair.

## 1 Introduction

Intestinal atresia is one of the most common congenital anomalies in neonates. As a general fact, they affect approximately 1 in 5000 live births. One of its unusual presentations is colonic atresia, which makes up to 1.8–15 % of reported intestinal atresia cases [1]. Colonic atresia can happen in any site of the colon, such as the ascending, transverse, or sigmoid colon [1]. Sigmoid atresia, for instance, can present as intestinal obstruction with abdominal distention within 24–48 h after birth, bilious vomiting, and failure to pass meconium [2]. Due to the fact that signs and symptoms of sigmoid atresia can resemble other diseases in neonates, diagnosis requires a high suspicion index and prompt medical evaluation and treatment [3]. This case report is written following the SCARE criteria [7].

## 2 Case presentation

A male newborn, three days old, was referred to our hospital from a rural hospital. The patient was born through normal vaginal delivery, is full-term, and weighs 2400 g. The patient had been experiencing bilious vomiting, inability to pass meconium, and abdominal distension for three days. Upon clinical examination, the patient had visible bowel loops and yellowish aspiration from the orogastric tube.

**Fig. 1:**
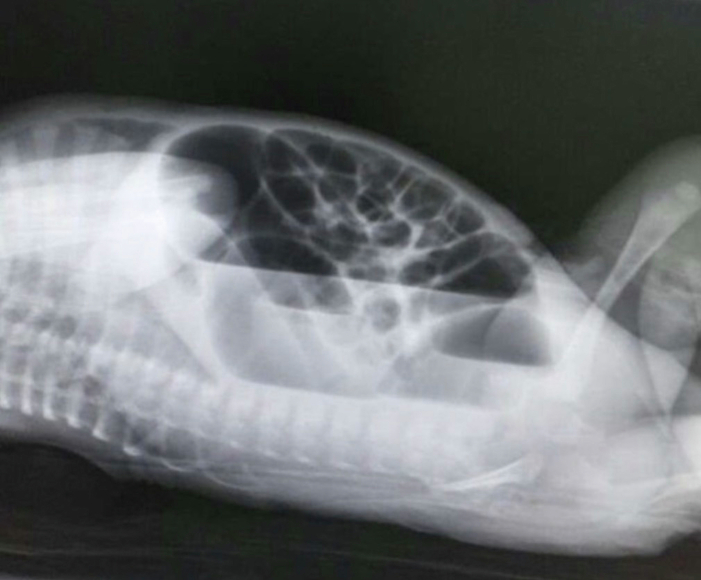
A cross-table abdominal x-ray revealed distended bowel loops with clearly defined air-fluid levels.

**Fig. 2:**
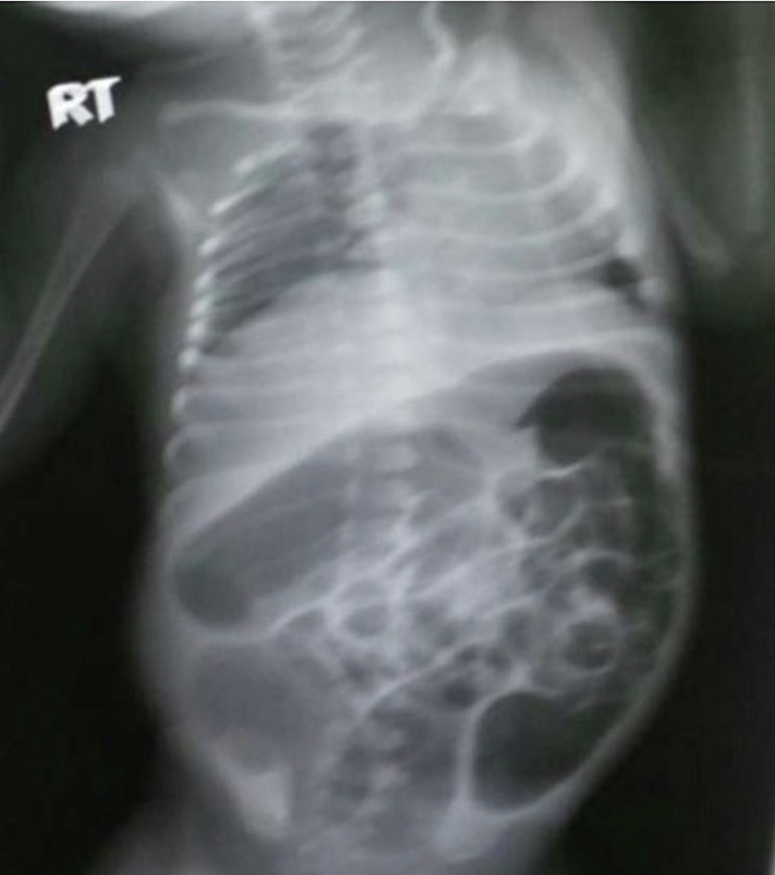
Anteroposterior abdominal x-ray revealed distended bowel loops with clearly defined air-fluid levels.

**Fig. 3:**
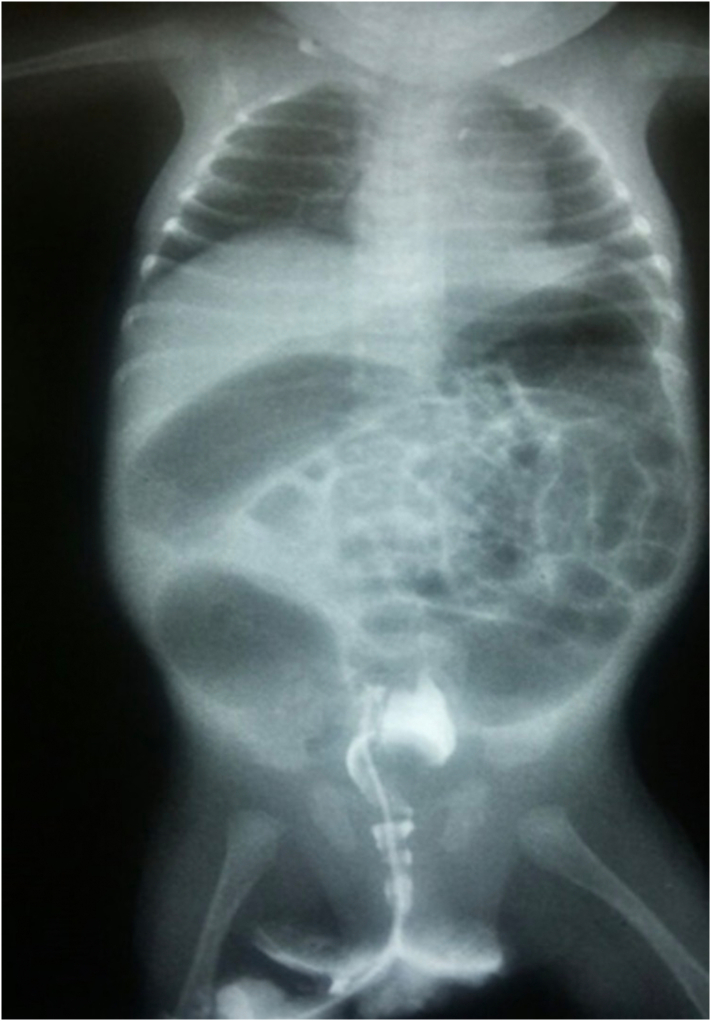
Contrast enema X-ray: A contrast enema showed resistance to the passage of the rectal tube and contrasted pooling in the rectosigmoid region (extravasation).

**Fig. 4:**
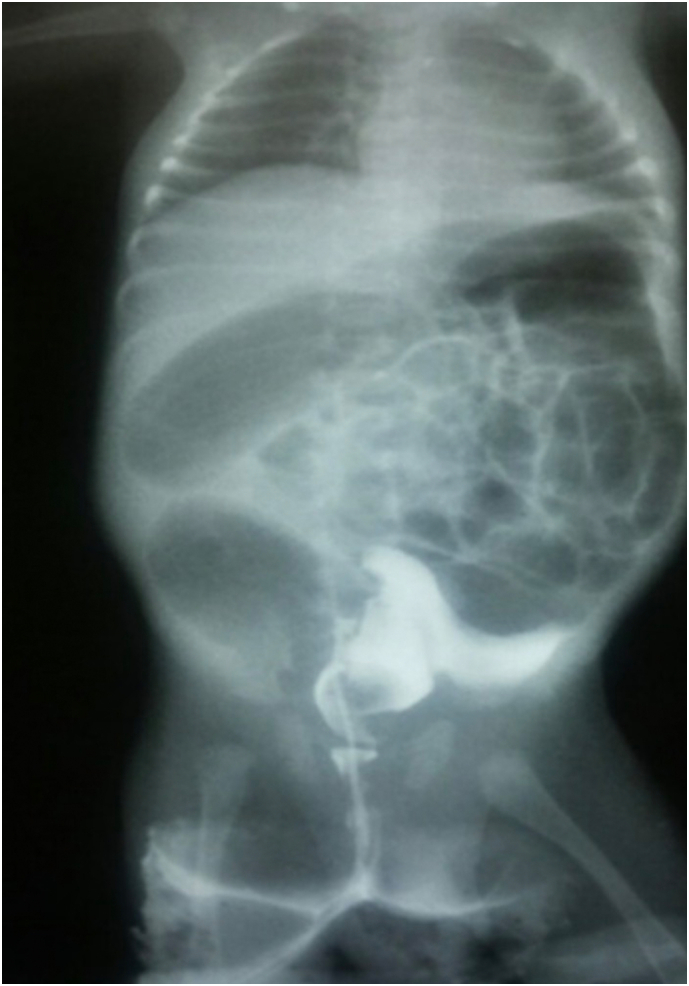
Contrast enema X-ray: A contrast enema showed resistance to the passage of the rectal tube and contrasted pooling in the rectosigmoid region (extravasation).

A laboratory workup was done and found to be unremarkable. A plain X-ray of the abdomen, both cross-table and anteroposterior supine, showed distended bowel loops with sharp air-fluid levels (Fig. 1, Fig. 2). During administering a contrast enema under fluoroscopy, resistance was encountered while attempting to pass the rectal tube. The contrast could not be injected beyond the rectosigmoid junction. Furthermore, contrast spillage from the anus around the tube was observed, indicating an intra-peritoneal perforation (Fig. 3, Fig. 4).

**Fig. 5:**
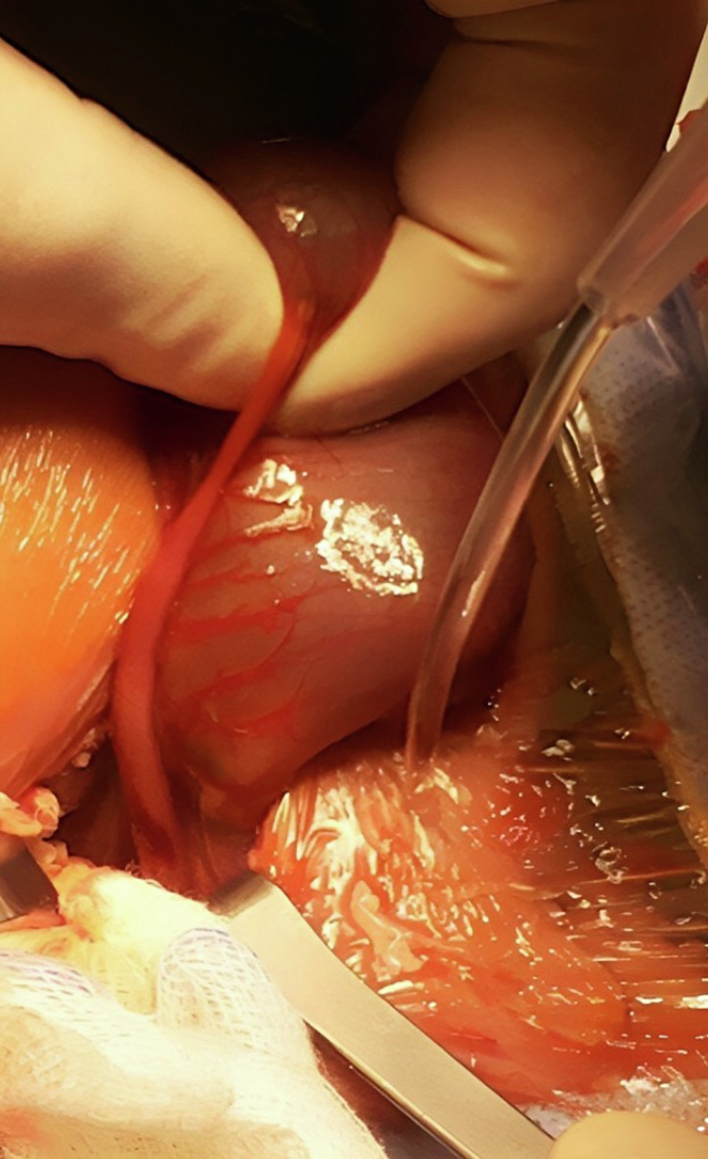
Intra-operative finding: A hugely dilated distal colon proximal to a blind end at the rectosigmoid junction. There was also a tiny perforation in the distal non-dilated part of the atretic segment with yellowish fluid (the injected dye).

**Fig. 6:**
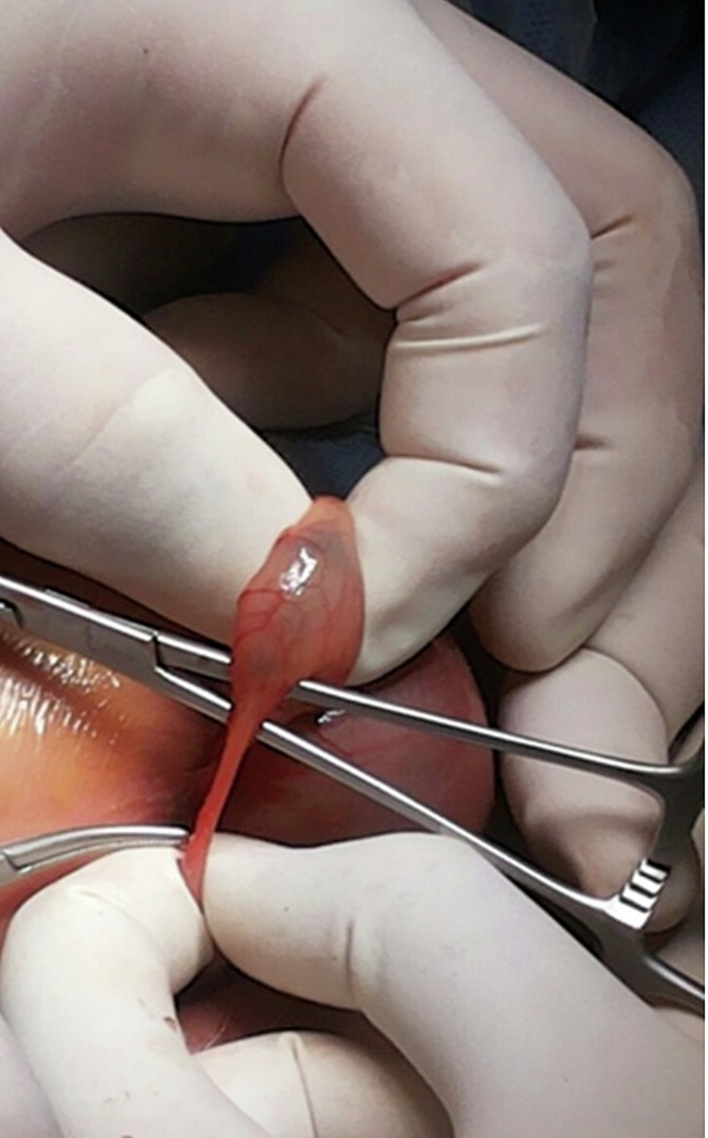
Intra-operative finding: A hugely dilated distal colon proximal to a blind end at the rectosigmoid junction. There was also a tiny perforation in the distal non-dilated part of the atretic segment with yellowish fluid (the injected dye).

A surgical procedure was planned for urgent laparotomy exploration. An oblique incision was made through the left iliac fossa, which revealed a hugely dilated distal colon proximal to the blind end. The atretic and rectosigmoid narrowing was also observed, along with a small perforation in the dilated proximal part of the atretic segment, which contained yellowish fluid (Fig. 5, Fig. 6). After resecting the atretic segment (about 5 cm) and thoroughly washing the peritoneal cavity, an end colostomy with a mucous fistula was made in the angles of the wound. The wound was then closed and dressed appropriately.

Histopathological examination for the resected part showed atretic colon, unremarkable colonic type tissue, with evident ganglion cells.

The patient had an uneventful postoperative period (discharged home on the fifth postoperative day), and follow-up reviews were unremarkable. Four months later, the patient was stable, growing well, and gaining weight, reaching 5 kg, so the colostomy was closed on an elective basis.

## 3 Discussion

Colonic atresia is rare, reporting an incidence of 1 in 20,000 live births [4]. It is thought to be caused by intrauterine vascular insults in the developing colon that can cause atresia, which can be associated with anorectal anomalies; however, sometimes, patients are diagnosed with sigmoid atresia, and such associations are not present [5]. The most common presentation of sigmoid atresia was bilious vomiting and abdominal distension in the first 24 h of life [2]. First, a radiography is done to diagnose it, and a significant intestine obstruction would be noticed. The diagnosis is then confirmed through a contrast enema. Also, a rectal biopsy is necessary to exclude Hirschsprung disease. An exploratory laparotomy is then done. The treatment is fully surgical. Primary anastomosis is done where a laparotomy, colostomy, and then a colo-colic anastomosis can also be done [6]. Uncomplicated colonic atresia can be managed by primary repair with little morbidity or with two-staged repair [1]. The literature provides evidence for the success of using a transanal approach to manage sigmoid atresia [7].

The case presented here is consistent with the typical presentation, diagnosis, and treatment of sigmoid atresia. The patient's clinical course was uncomplicated in the second stage (colostomy closure), and he was discharged home on the fifth postoperative day with good passage of stool. Feeding started on the third postoperative day. There were no associations with the atresia. This case report adds to the growing body of literature on sigmoid atresia and highlights the importance of early diagnosis and treatment.

The findings of this case report suggest that sigmoid atresia is a rare but treatable congenital anomaly. Early diagnosis and treatment are essential to achieve a good outcome.

## 4 Conclusion

Sigmoid atresia as an entity requires extreme caution from the treating physician, as this anomaly can present with a different set of anomalies or can mimic Hirschsprung's disease. In our case, the patient had a classical presentation of sigmoid atresia with an uncomplicated postoperative course. Our case highlights high clinical suspicion, and radiographic findings are clues for diagnosis. Early diagnosis can result in optimal outcomes with a better prognosis.

## Consent

Written informed consent was obtained from the patients parents/legal guardner for publication and any accompanying images. A cope of the written consent is available for review by the Editor-in-Chief of this journal on request.

## Ethical approval

This is a case report and according DHHS is a medical/educational activity that does not meet the DHHS definition of “research”.

## Funding

Not applicable.

## Author contribution

A. Sayed, K. Alnajjar, O. almatrafi, M. Aldamouni participated in writing the background with significance, case presentations, discussion, and conclusion, read and approved the final manuscript.

A. Abokrecha, A. Sayed helped draft the manuscript, read, and agree on the final manuscript, and formatted the manuscript according to the journal's guidelines.

A. Abokrecha, A. Sayed, supervising the overall research, preparing the manuscript reading, and approving the final manuscript.

## Guarantor

Ahmed Abokrecha accepts full responsibility for the work and/or the conducted of the study, had access to the data and controlled the decision to publish.

## Conflict of interest statement

The authors declare that the research was conducted in the absence of any commercial or financial relationships that could be construed as a potential conflict of interest.
